# Chloroplast genome and haplotype relationships unravel the genetic introgression and complex evolutionary history of East Asian *Rosa* section *Synstylae* roses (Rosaceae)

**DOI:** 10.1186/s40529-025-00466-y

**Published:** 2025-06-13

**Authors:** Ji-Hyeon Jeon, Masayuki Maki, Yu-Chung Chiang, Seung-Chul Kim

**Affiliations:** 1 Department of Biological Sciences, Sungkyunkwan University, Suwon, 16419 Republic of Korea; 2 Department of Ecological Developmental Adaptability Life Sciences, Tohoku University, Sendai, 980-8577 Japan; 3 Department of Biological Sciences, National Sun Yat-sen University, Kaohsiung, 80424 Taiwan

**Keywords:** *Rosa*, Section *Synstylae*, Rosaceae, Eastern Asiatic floristic region, Genetic introgression, Chloroplast capture, Chloroplast genome, Phylogeny, Haplotype analysis

## Abstract

**Background:**

The section *Synstylae* of the genus *Rosa* (Rosaceae) is predominantly distributed across the Eastern Asiatic Floristic Region and is characterized by increased species diversity and natural hybrids. These characteristics render species within this section exemplary for studying phenotypic variability and easy crossbreeding, which hold potential for advancements in the rose-breeding industry. However, genetic introgression and hybridization have posed challenges to our understanding of their phylogenetic relationships. Despite recurrent interspecific introgression, chloroplast DNA can still aid in phylogenetic inference within the section *Synstylae* due to its uniparental inheritance and high conservation.

**Results:**

Phylogenetic inferences and haplotype network analysis identified seven distinct chloroplast haplotype groups within the East Asian *Synstylae*. Clear differentiation was observed between the chloroplast haplotypes of the Sino-Himalayan series *Brunonianae* and Sino-Japanese series *Multiflorae* lineages. The chloroplast haplotypes within each lineage aligned more closely with geographic gradients than with species boundaries. Consequently, various chloroplast haplotypes were shared among Sino-Japanese *Synstylae* species with broader distributions, whereas unique haplotypes were found in the species with restricted distribution ranges. Similarly, geographically specific haplotype groups were identified in the Japanese Archipelago, Taiwan, and Eastern China of the Sino-Japanese Subregion, respectively.

**Conclusions:**

The chloroplast genomes of Sino-Japanese *Synstylae* species may have diverged along geographic gradients, influenced by the geographical and ecological complexity of East Asia and the climate oscillations during the Pleistocene. The recurring cycles of fragmentation and rejoining in Sino-Japanese *Synstylae* populations have allowed founder effects and genetic drift to drive divergence and diversification of their chloroplast genomes along these geographic gradients. The substantial incongruence between the chloroplast and nuclear phylogenies evidenced the prevalent genetic introgression within the Sino-Japanese *Synstylae* lineage. Additionally, two putative hybrid speciation events highlighted the role of genetic introgression in species diversification of the East Asian *Synstylae* lineage. This study substantiates the value of chloroplast genomes in elucidating genetic introgression and the unique evolutionary history of recently diverged and closely related East Asian *Synstylae* species.

**Supplementary Information:**

The online version contains supplementary material available at 10.1186/s40529-025-00466-y.

## Background

The genus *Rosa* (Rosaceae) comprises over a hundred wild rose species across the temperate and subtropical forests of the Northern Hemisphere (Rehder 1940; Yu and Ku 1985; Wissemann 2003), as well as more than 30,000 modern rose cultivars cherished worldwide (Young et al. 2007; Leus et al. 2018). East Asian wild roses have been pivotal in breeding modern roses, with four of the seven major wild progenitors originating from East Asia (*Rosa chinensis* var. *spontanea*, *R. odorata* var. *gigantea*, *R. lucieae*, and *R. multiflora*; Wylie 1954), by contributing to attributes such as flowering, petal color, fragrance, stress resistance, and ease of crossbreeding (Van Fleet 1908; Wylie 1954). In the widely accepted infrageneric classification of the genus *Rosa* (Rehder 1940; Yu and Ku 1985; Wissemann 2003), these wild progenitors are classified into two closely related sections: *Chinenses* (*R. chinensis* var. *spontanea* and *R. odorata* var. *gigantea*) and *Synstylae* (*R. lucieae* and *R. multiflora*), of which species predominantly occur in East Asia. Notably, the East Asian *Synstylae* illustrates the increased species diversity and natural hybrids (Wissemann and Ritz 2005; Ohba et al. 2007; Zhu and Gao 2015; Zhu et al. 2015; Ohba and Akiyama 2019; Debray et al. 2022; Zhang et al. 2024; Jeon et al. 2025), which holds potential for the advancements in the rose-breeding industry through improved understanding of their genetic background (De Cock et al. 2007).

The East Asian *Synstylae* species are distributed across the Eastern Asiatic Floristic Region (sensu Takhtajan 1978), which is a conventionally well-delineated floristic region encompassing the subtropical and temperate forests of East Asia, established through a series of floristic studies (Grisebach 1872; Drude 1890; Diels 1901; Engler 1903; Good 1947; Takhtajan 1978). The Eastern Asiatic Floristic Region is subdivided into two subregions: i) the Sino-Himalayan Subregion, which ranges from the southeastern slopes of the Tibetan Plateau to the mountain ranges of Southwestern China; and ii) the Sino-Japanese Subregion, which includes subtropical China, Northeastern China, the Japanese Archipelago, Korean Peninsula, Russian Manchuria, and Taiwan (see Fig. S1; Takhtajan 1978; Wu 1998; Qiu et al. 2011). East Asian *Synstylae* species exhibit distinct distributions within the Eastern Asiatic Floristic Region. For instance, *Rosa brunonii*, *R. filipes*, *R. helenae*, *R. lichiangensis*, *R. longicuspis*, and *R. soulieana* occur in the Sino-Himalayan Subregion, whereas *R. lucieae*, *R. maximowicziana*, *R. multiflora*, *R. onoei*, *R. paniculigera*, *R. pricei*, *R. sambucina*, *R. taiwanensis*, and *R. transmorrisonensis* occur in the Sino-Japanese Subregion (Table S1). These different geographical distributions corroborate the morphological infra-sectional classification of the section *Synstylae*, which includes the series *Brunonianae* with entire stipule margins and the series *Multiflorae* with serrate/pectinate stipule margins (Yu and Ku 1985). Generally, *Brunonianae* species are found in the Sino-Himalayan Subregion, whereas *Multiflorae* species are found in the Sino-Japanese Subregion (see Table S1). East Asian *Synstylae* species have rapidly diversified within the Sino-Japanese Subregion due to its geographical and ecological complexity (Jeon et al. 2025). However, their rapid diversification has resulted in close genetic and phylogenetic relationships, leading to recurrent genetic introgression among them (Zhu et al. 2015; Debray et al. 2022; Jeon et al. 2025).

Roses are known for their self-incompatibility and interspecific hybridization, both spontaneous and artificial, which enhance phenotypic variability and genomic heterozygosity (Gudin 2003; Bendahmane et al. 2013; Raymond et al. 2018). Hybridization-mediated genetic introgression can provide opportunities for the increased fitness and emergence of new lineages (Rieseberg et al. 2003; Twyford and Ennos 2012). Nevertheless, genetic introgression and hybridization have hindered our understanding of phylogenetic relationships and species boundaries within the genus *Rosa* (Wissemann and Ritz 2005; Bruneau et al. 2007; Fougère-Danezan et al. 2015; Zhu et al. 2015; Debray et al. 2022). Although chloroplast DNA sequences are advantageous for phylogenetic inference in lineages with interspecific introgression due to their uniparental inheritance and high conservation (Palmer et al. 1988; Cronn et al. 2008), chloroplast genomic phylogenetic inference for three East Asian *Synstylae* species from distinct florae in the Sino-Japanese Subregion failed to resolve their relationships (*R. lucieae* from Korean-Japanese flora, *R. maximowicziana* from Amur flora, and *R. multiflora* from Liaoning-Shandong flora; Chang et al. 2011; Kim and Chang 2015; Jeon and Kim 2019). Jeon and Kim (2019) suggested that these close relationships may be due to insufficient evolutionary time for chloroplast haplotype divergence, or chloroplast capture resulting from interspecific introgression along geographic gradients.

In the present study, we revisited the chloroplast phylogeny of East Asian *Rosa* sect. *Synstylae* to explore phylogenetically distinct haplotypes using chloroplast genomic sequence data. We further looked into phylogenetic relationships and divergence of chloroplast haplotype lineages within the East Asian *Synstylae*, by comparing them with the species phylogeny inferred from nuclear genomic data in an earlier phylogenetic study (Jeon et al. 2025). We then analyzed the chloroplast haplotypes of *Synstylae* lineages occurring in the Sino-Japanese Subregion (hereafter, the Sino-Japanese *Synstylae*; see Table S1) to clarify their phylogeographic relationships and evolutionary history under potential genetic introgression using highly variable chloroplast genetic regions. Consequently, we aimed to elucidate the impact of genetic introgression on divergence and diversification of the Sino-Japanese *Synstylae* species, and substantiate the utility of chloroplast sequences in identifying introgression among closely related taxa.

## Materials and methods

### Taxon sampling and DNA isolation

For subsequent chloroplast haplotype analyses, we retrieved dried leaf materials of 54 wild accessions from an earlier nuclear phylogenetic study of the East Asian *Rosa* sect. *Synstylae* roses to compare their phylogenies and evolutionary histories of chloroplast and nuclear DNAs (Table S2; Jeon et al. 2025). Voucher specimens of the accessions were deposited in the Ha Eun Herbarium at Sungkyunkwan University (SKK), the Herbarium of Arnold Arboretum (A), and the Herbarium of Taiwan Forestry Research Institute (TAIF). The sampling included accessions from nine of 14 well-understood Sino-Japanese *Synstylae* species, representing geographical and environmental diversity within the Eastern Asiatic Floristic Region (Yu and Ku 1985; Ohba 1993; Kim 2022; Hung and Wang 2022). To identify diverse haplotypes, four or more accessions per species from various localities were retrieved for the present study including the following chloroplast genomic accessions. Genomic DNA (gDNA) was isolated from the dried leaf tissues using the Exgene Plant SV Mini Kit (GeneAll Biotechnology Co., Ltd., Seoul, Korea). We followed either the manufacturer’s instructions or a modified protocol developed by Costa and Roberts (2014) to isolate gDNAs from the leaf materials.

### Chloroplast genome assembly and marker selection

We retrieved 30 chloroplast genome accessions from NCBI GenBank, including section *Synstylae* accessions and outgroup accessions from closely related infrageneric groups within the genus *Rosa* (subg. *Rosa* sect. *Chinenses*, subg. *Rosa* sect. *Microphyllae*, and subg. *Hulthemia*) and the closely related genus *Fragaria* (Table S3) for subsequent chloroplast phylogenomic and haplotype analyses. In addition, we retrieved five whole-genome sequencing reads from the NCBI Sequence Read Archive (SRA) to assemble novel chloroplast genome sequences of Japanese and Taiwanese *Synstylae* species (*Rosa onoei*, *R. paniculigera*, *R. sambucina*, *R. taiwanensis*, and *R. transmorrisonensis*; Table S4).

Each set of sequencing reads retrieved from the SRA was preprocessed to trim sequencing adapters, polymerase chain reaction (PCR) dimers, and low-quality sequences using BBDuk v38.26 (Bushnell 2014). Subsequently, each set of trimmed sequence reads was assembled *de novo* into chloroplast genomic sequences using NOVOPlasty v4.2 (Dierckxsens et al. 2017), with the *k*-mer length of 33 and utilizing the *matK* sequence of the reference chloroplast genome of *R. lucieae* (NCBI Gene ID: 39119432) as an assembly seed. The genomic structure and functional genes of each chloroplast genome were annotated using GeSeq (Tillich et al. 2017), incorporating predictions from Chloë v0.1.0 (Small et al., 2021) and tRNAscan-SE v2.0.7 (Chan et al. 2021), and gene alignments referenced from the chloroplast genome of *Rosa roxburghii* (sect. *Microphyllae*; NCBI RefSeq accession: NC_032038.1; Wang et al. 2018) using BLAT (Kent 2002). The genomic structure and gene annotations were visualized on a graphical map using OGDRAW v1.3.1 (Fig. S2; Greiner et al. 2019). The chloroplast genome assemblies generated in this study were deposited in GenBank (Table S4).

To pinpoint efficient genetic markers in chloroplast haplotype analyses of the East Asian *Synstylae*, we explored chloroplast genomes of closely related Sino-Japanese series *Multiflorae* species. Considering the substantially low mutation rates in gene products of the *Rosa* chloroplast genomes (Jeon and Kim 2019; Zhang et al. 2022), the occurrence of lineage-specific mutations in multiple genic regions affecting adaptation and selection is instrumental for delineating distinct chloroplast lineages. Additionally, given that introns and intergenic spacers tend to accumulate homoplasious and parsimony-uninformative variation despite their advantage of high variability, we focused on the coding DNA sequences (CDSs) of genic regions to demarcate robust chloroplast haplotype groups characterized by conserved and homologous mutations within the East Asian *Synstylae*. Within the alignment of 15 chloroplast genome sequences using MAFFT v7.480 (Katoh and Standley 2013), four highly variable genic regions (*ndhF*, *rbcL*, *rpoC1*, and *ycf1*) were selected based on the number of parsimony-informative sites and nucleotide diversity (π) (Fig. S3). Primer pairs for each chloroplast genetic marker were designed specifically for *Rosa* species using Primer3 v2.3.7 (Table S5; Untergasser et al. 2012).

### DNA amplification and sequencing

The partial CDSs of the four highly variable chloroplast genic regions were amplified using PCR to identify the chloroplast haplotypes of the Sino-Japanese *Synstylae* accessions collected in this study. Each reaction included 0.2 µM of each primer, 2 mM MgCl_2_, 0.2 mM of each dNTP, and 1.25 units of Inclone *Taq* DNA polymerase (Inclone Biotech Co., Ltd., Seoul, Korea). The cycling parameters were (1) 95 ºC initial denaturation for 2 min, (2) 35 cycles of 95 ºC denaturation for 20 s, 56 ºC annealing for 40 s, and 72 ºC extension for 1 min, and (3) 72 ºC final extension for 5 min. The PCR amplicons were purified using the Inclone Gel & PCR purification kit (Inclone Biotech Co., Ltd.) following the manufacturer’s instructions, and then Sanger sequenced by GenoTech Corp. (Daejeon, Korea). The genetic sequences generated in this study have been deposited in GenBank (Table S2). For accessions with existing genomic sequences, the genetic sequences of the four highly variable genic regions were extracted using in silico PCR, with primer pairs mapped to each genomic sequence using Primer3 implemented in Geneious R10 v10.2.3 (Biomatters, Ltd., Auckland, New Zealand).

### Chloroplast phylogenomic inference

A total of 33 chloroplast genome sequences were aligned using MAFFT to understand the chloroplast genome-based phylogenetic relationships and delimit the haplotype groups of East Asian section *Synstylae* species. The protein-coding and non-coding-RNA sequences from the chloroplast genome sequence alignment were subjected to maximum-likelihood (ML) inference using IQ-TREE v2.3.4 (Minh et al. 2020) to resolve the chloroplast genome-based phylogenetic relationships among East Asian *Synstylae* species. ML inference was analyzed with the TVM + F + I substitution model selected by ModelFinder (Kalyaanamoorthy et al. 2017), and tree robustness was assessed through bootstrap approximation with 5000 replicates using UFBoot2 (Hoang et al. 2018).

The divergence times of chloroplast haplotype groups within the section *Synstylae* were estimated using BEAST2 v2.6.7 (Bouckaert et al. 2019). An alignment subset of 22 chloroplast genome sequences, including one representative genome sequence per species, was analyzed for divergence time estimation with the GTR + F + I site model specified by ModelFinder, a strict molecular clock model, the birth-death model tree prior, and three calibration priors. Molecular clock calibration included one fossil calibration based on the fossil of *Rosa germerensis* (55.8–48.6 Mya; Idaho, USA; Edelman 1975), and two secondary calibrations with log-normal priors for the divergence points of the subgenus *Rosa*, and between the genera *Rosa* and the *Fragaria*, based on the divergence time estimation from their nuclear genomic sequences (Jeon et al. 2025). Two MCMC chains were run for 100,000,000 generations for divergence time estimation, with sampling of parameters and trees every 1000 generations. Chain convergence and the effective sample size (ESS) of the posterior were monitored using Tracer v1.7.2 (Rambaut et al. 2018), and a maximum clade credibility tree was derived using TreeAnnotator v2.6.7 (Bouckaert et al. 2019), with discarding the first 10% of trees as post-burn-in.

### Chloroplast haplotype analyses

The haplotype sequences of the 83 East Asian *Synstylae* and *Chinenses* accessions for each highly variable chloroplast genic region were aligned using the Geneious Aligner in Geneious R10. The alignments of the four highly variable chloroplast genic regions were then concatenated for subsequent analyses. Particularly, the sequence alignment of the Sino-Japanese *Synstylae* species was used in haplotype network analysis with the statistical parsimony method using TCS v1.23 (Clement et al. 2000). Phylogenetic relationships among East Asian *Synstylae* species were inferred by ML inference and Bayesian inference (BI) using IQ-TREE and MrBayes v3.2.7 (Ronquist et al. 2012) respectively, with each genic region partitioned to specify a distinct codon substitution model using ModelFinder (Table S5). BI was analyzed with four MCMC chains run for 10,000,000 generations, with sampling of parameters and trees every 1000 generations. Tree robustness of ML inference was assessed through bootstrap approximation with 5000 replicates using UFBoot2. The mixing and convergence of the MCMC in BI were assessed using the ESS and the potential scale reduction factor (PSRF) (Ripley 1987; Gelman and Rubin 1992; Table S6). Haplotype diversity was calculated for each region of the Eastern Asiatic Floristic Region, and mutation neutrality was evaluated using Tajima’s *D* (Tajima 1989) and Fu’s *F*_*S*_ (Fu 1997) in Arlequin v3.5.2.2 (Excoffier and Lischer 2010). Given the regional prevalence of interspecific gene flow within the East Asian *Synstylae* (Ohba et al. 2007; Jeon and Kim 2019; Ohba and Akiyama 2019; Zhang et al. 2024), we also calculated Tajima’s *D* and Fu’s *F*_*S*_ for each geographic region, without considering taxonomic designation.

## Results

### Chloroplast genome assembly and phylogenomic inference

Each set of high-throughput sequencing reads for the five Japanese and Taiwanese species was assembled into a complete circular chloroplast genomic sequence, with coverage depth ranging from 274.0 to 2562.9× and the length from 156,487 to 156,604 bp (Table S4). Each chloroplast genome displayed a quadripartite structure, in which a large single-copy region (LSC; 85,635–85,741 bp) and a small single-copy region (SSC; 18,740–18,765 bp) were separated by two inverted repeats (IRs; 26,049–26,058 bp) (Fig. S2). A total of 112 unique genes were identified within each genome, including 78 protein-coding, 30 tRNA, and four rRNA genes (Table S4).

**Fig. 1:**
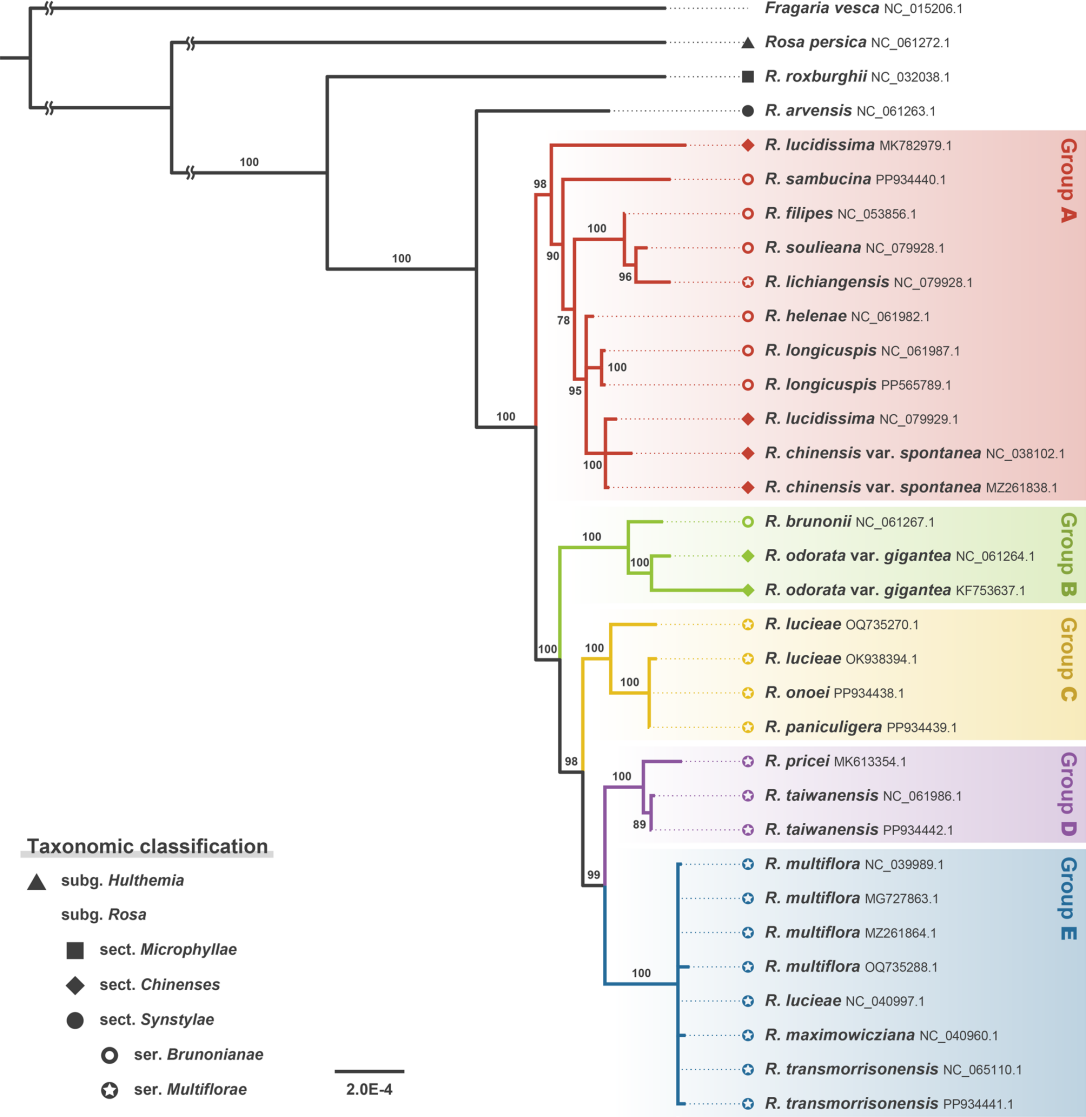
Maximum-likelihood phylogenetic tree of chloroplast haplotypes of the East Asian *Synstylae* inferred from chloroplast genome-wide protein-coding and non-coding-RNA sequences. Numbers on branches indicate the bootstrap branch support. Tip labels are species names of accessions, followed by accession IDs. Tip shapes indicate the infrageneric taxonomic classification of accessions in the genus *Rosa*. The colors of branches, tips, and tip-label blocks represent the chloroplast haplotype groups

Including these five chloroplast genomic sequences, ML phylogenomic inference of the chloroplast genomes of East Asian *Synstylae* taxa identified five distinct and robust chloroplast haplotype groups, each with strong branch support (98–100% bootstrap support values; Fig. 1). Accessions from the section *Chinenses*, which is the closest relative to *Synstylae*, were nested in the East Asian *Synstylae* clade. Haplotype groups A and B included the accessions of the Sino-Himalayan or series *Brunonianae* species, and haplotype groups C, D, and E included the Sino-Japanese accessions of the series *Multiflorae* species (see Table S1). The *Chinenses* accessions were polyphyletic and separated into two distinct haplotype groups, with *R. chinensis* var. *spontanea* and *R. lucidissima* in haplotype group A, and *R. odorata* var. *gigantea* in group B. Similarly, the Sino-Himalayan *Brunonianae* accessions were polyphyletic, with *R. brunonii* solely in haplotype group B, whereas the Sino-Japanese *Multiflorae* accessions were monophyletic (haplotype groups C–E). Japanese-endemic species (*R. onoei* and *R. paniculigera*) shared haplotype group C, and Taiwanese-endemic *R. transmorrisonensis* fell within group E, whereas other Taiwanese-endemic species (*R. pricei* and *R. taiwanensis*) grouped within group D.

**Fig. 2:**
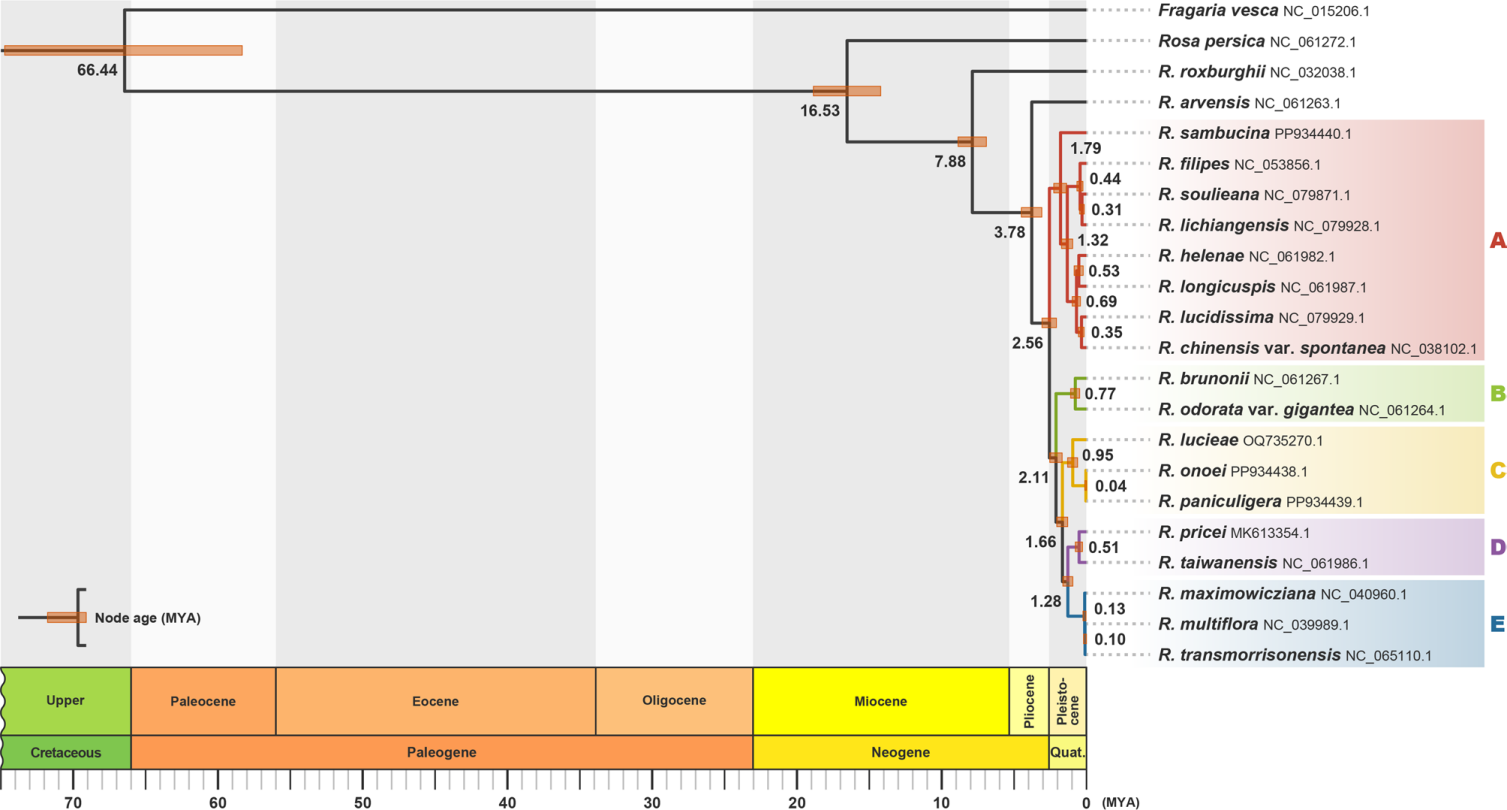
Chronogram showing divergence times of chloroplast haplotypes of the East Asian *Synstylae*. Numbers beside nodes indicate the node ages, and orange bars on the nodes represent 95% confidence intervals. Tip labels are species names of accessions, followed by accession IDs. The colored tip-label blocks represent the chloroplast haplotype groups

Molecular dating analysis estimated the crown age of the chloroplast lineage of sections *Synstylae* and *Chinenses* (including European *Rosa arvensis*) at 3.78 Mya, whereas the crown age of East Asian chloroplast lineage was estimated at 2.56 Mya (Fig. 2). The divergence times of East Asian haplotype groups were estimated between 2.56 and 1.28 Mya, with crown ages at 1.79–0.13 Mya, suggesting their divergence during the Pleistocene. In molecular dating, an ESS of 66,096.1 for the posterior indicated sufficient mixing of the MCMC.

### Haplotype network, diversity, geographical distribution and phylogeny

**Fig. 3:**
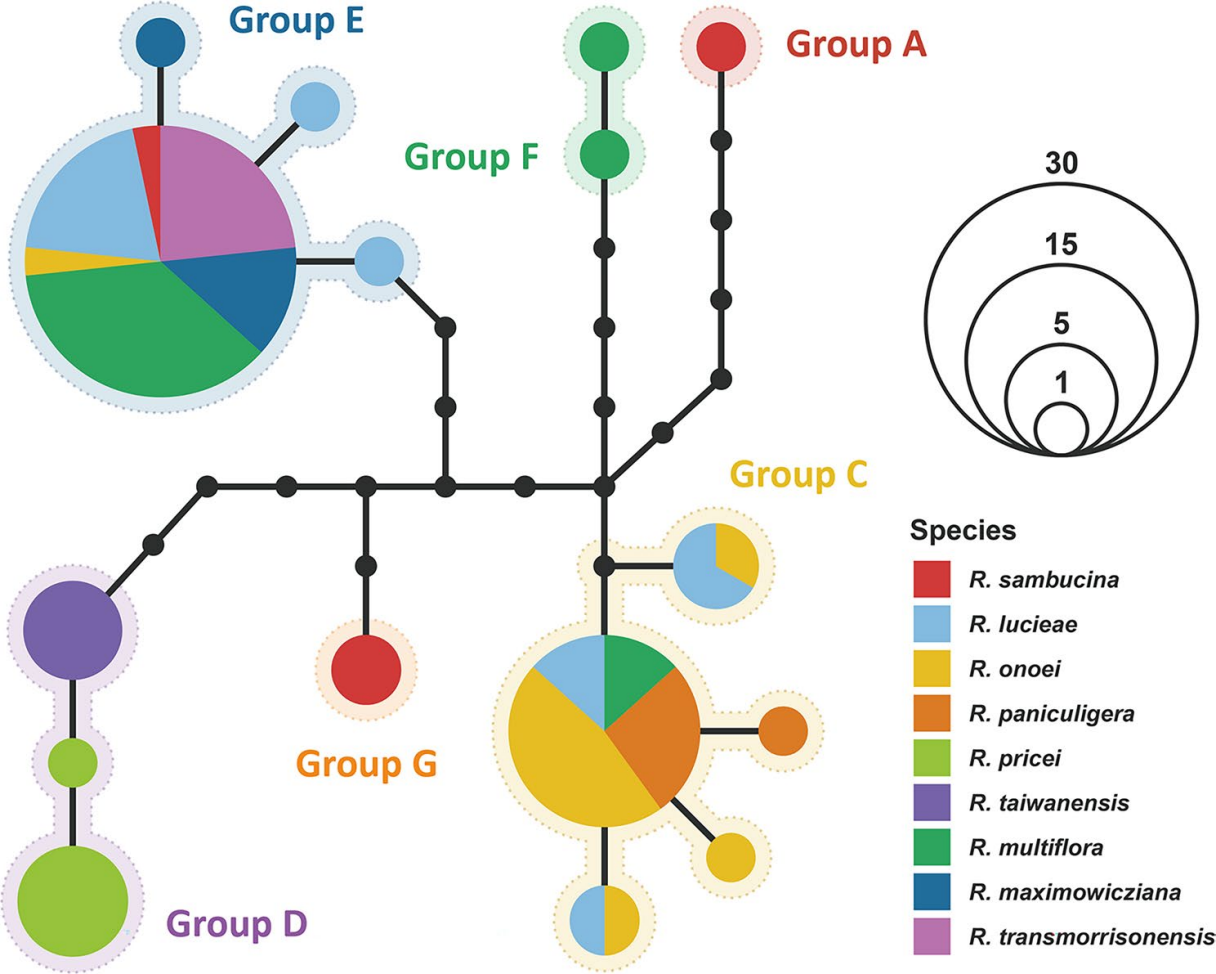
Statistical parsimony haplotype network of Sino-Japanese *Synstylae* inferred from the four highly variable chloroplast genic regions. The colored pie charts indicate the haplotypes, and the closed circles on the internal nodes indicate the putative missing intermediate haplotypes. The colors of the slices in the pie charts represent the species, and the sizes of the pie charts are proportional to the abundance of the haplotypes. Dotted border lines around the haplotype pie charts indicate haplotype groups

**Fig. 4:**
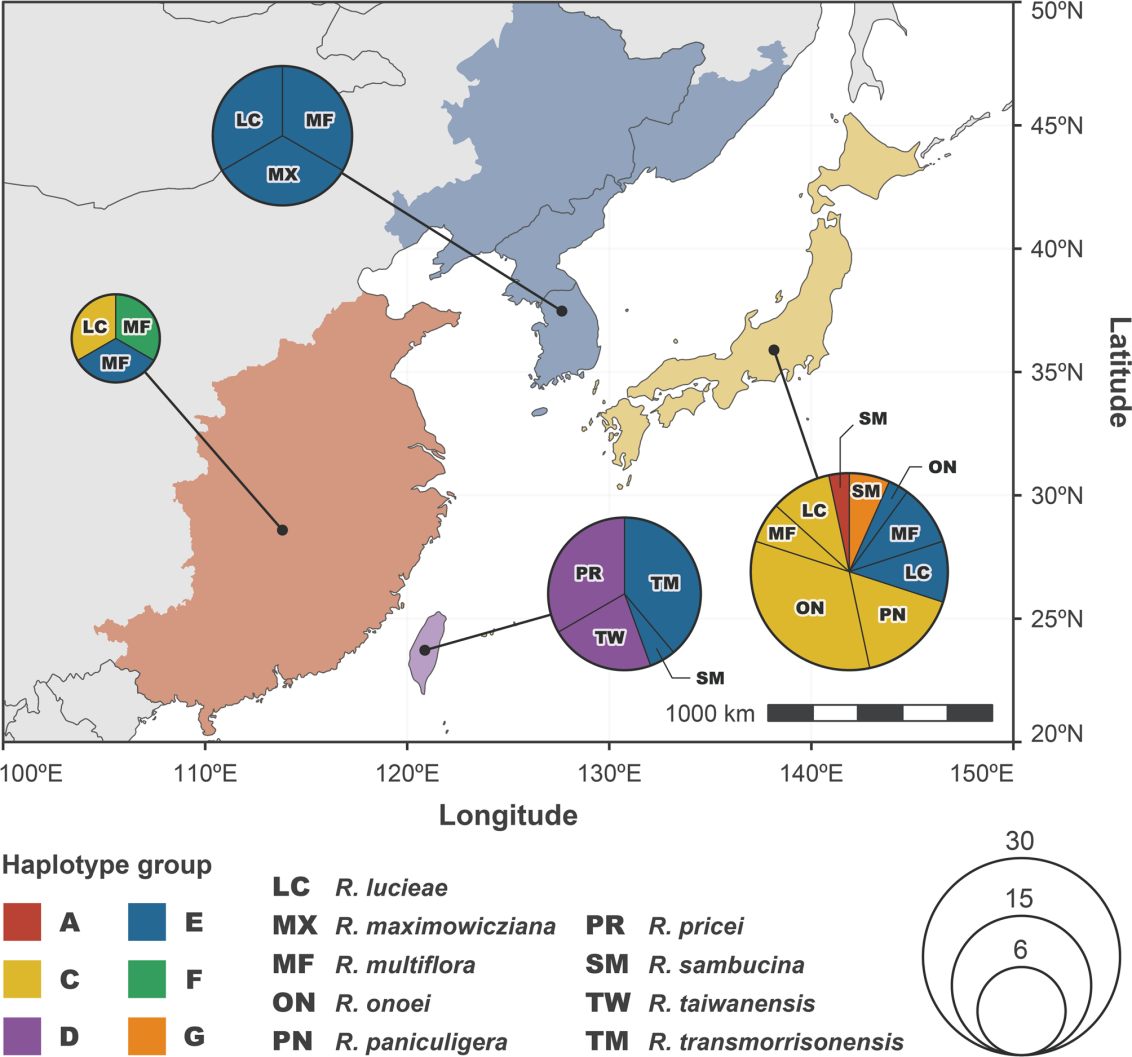
Map of the geographical distribution of chloroplast haplotypes of the Sino-Japanese *Synstylae* species. Each colored region represents a distinct locality in the Sino-Japanese Subregion: South Central-Eastern China (orange), Taiwan (purple), the Korean-Manchurian region (blue), and the Japanese Archipelago (yellow). Pie charts represent the haplotype ratios in the respective regions. The colors of the slices in the pie charts represent haplotype groups, and the sizes of the pie charts are proportional to the number of accessions. Two-letter abbreviations in the pie charts indicate the species names

Haplotype network analysis of the Sino-Japanese *Synstylae* accessions using sequence data from the four highly variable chloroplast genic regions identified six distinct haplotype groups, including two novel groups (F and G) that were not detected in the chloroplast phylogenomic inference (Figs. 1 and 3). Phylogenetic inference of haplotypes from the East Asian *Synstylae* accessions further supported the distinct phylogenetic lineage of each haplotype group, with inferring identical tree topologies from ML and BI analyses, although group A appeared to be paraphyletic and polytomous (Fig. S4). The most prevalent haplotype groups (i.e., groups C, D, and E) were shared across various Sino-Japanese *Synstylae* species (Fig. 3; Fig. S4). For example, group E was notably shared by six of the nine Sino-Japanese *Synstylae* species, with Japanese-endemic *R. paniculigera* and Taiwanese-endemic *R. pricei* and *R. taiwanensis* being exceptions. The Sino-Japanese *Synstylae* species distributed in restricted areas exhibited unique haplotypes, whereas species with broader distributions varied in their chloroplast haplotypes (Figs. 3 and 4). For example, most accessions of Japanese-endemic *R. onoei* and *R. paniculigera* shared haplotype group C (except for one *R. onoei* accession in group E), accessions of Taiwanese-endemic *R. pricei* and *R. taiwanensis* shared group D, and accessions of Taiwanese-endemic *R. transmorrisonensis* and Northeastern Asian *R. maximowicziana* shared group E. In contrast, widely distributed *R. lucieae*, *R. multiflora*, and *R. sambucina* included accessions with a variety of haplotypes.

The geographical distribution of chloroplast haplotypes within the Sino-Japanese *Synstylae* lineage exhibited two different patterns. Whereas haplotype group E was broadly distributed across the Sino-Japanese Subregion, other groups were restricted to specific areas (Fig. 4). For example, accessions in haplotype group C were predominantly found in the Japanese Archipelago, and those in group D were exclusively found in Taiwan. Unique haplotype groups were found in most geographic areas of the Sino-Japanese Subregion (e.g., haplotype group D in Taiwan, group F in Eastern China, and group G in the Japanese Archipelago; see Fig. 4). Multiple haplotype groups were found in each of these areas, with haplotype diversity of 0.7124–0.9524 and nucleotide diversity of 0.0010–0.0020 (Table S7). However, no specific haplotype group was identified in the region encompassing Korean Peninsula, Northeastern China, and Russian Manchuria (the blue-colored region in Fig. 4; hereafter, the Korean-Manchurian region), where only accessions in haplotype group E were found with low haplotype diversity of 0.3714 and nucleotide diversity of 0.0001 (Table S7). Neutrality tests (Tajima’s *D* and Fu’s *F*_*S*_ statistics) of the East Asian regions indicated wide ranges of Tajima’s *D* values of − 1.6850–2.7430 (*p* = 0.0268–0.9996) and Fu’s *F*_*S*_ values of − 2.3691–5.8000 (*p* = 0.0035–0.9820; Table S7).

## Discussion

### Divergence of chloroplast genomes within the East Asian *Synstylae*

The phylogenetic tree inferred from chloroplast genomes identified distinct haplotype groups within the East Asian *Synstylae*, and haplotype network analysis also delineated these groups, separated by several missing intermediate haplotypes (Figs. 1 and 3). Estimated divergence times of the haplotype groups ranged from 2.56 to 1.28 Mya (Fig. 2), which overlapped with species divergence times of the East Asian *Synstylae* (1.97–0.73 Mya; Jeon et al. 2025). This contemporaneous divergence of chloroplast haplotype groups and species of the East Asian *Synstylae* reduces the plausibility of one of the hypotheses proposed by Jeon and Kim (2019), which suggested that chloroplast genomes of the East Asian *Synstylae* species failed to diverge due to insufficient evolutionary time since their origin. Instead, the shared haplotypes among accessions of different species support the alternative hypothesis, in which chloroplast capture through interspecific introgression blurred phylogenetic relationships among East Asian *Synstylae* species. According to this hypothesis, chloroplast divergence likely followed geographic gradients rather than taxonomic designation. Consistent with this, our results indicated the probable geographical divergence of chloroplast haplotypes within the Sino-Japanese *Synstylae*. Although major haplotype groups C, D, and E encompassed multiple species, group C was predominantly found in the Japanese Archipelago, group E in the Korean-Manchurian region, and group D was restricted to Taiwan (Figs. 3 and 4). Additionally, minor haplotype groups F and G were confined to Eastern China and the Japanese Archipelago, respectively (Fig. 4). Nevertheless, the former hypothesis remains applicable to recently diverged species lineages that emerged after haplotype divergence. For example, *R. paniculigera* and *R. onoei* (estimated divergence time: 0.73 Mya) predominantly shared a single haplotype in group C, whereas *R. transmorrisonensis* and *R. multiflora* (estimated divergence time: 0.88 Mya) shared an identical haplotype in group E (Fig. 3; Jeon et al. 2025). The diversification of East Asian *Synstylae* species has been associated with migration from the Sino-Himalayan Subregion to the Sino-Japanese Subregion, alongside the geographical and ecological complexity of East Asia and climate oscillations during the Pleistocene, especially within the Sino-Japanese Subregion (Qian and Ricklefs 2000; Harrison et al. 2001; Jeon et al. 2025). Given their divergence times and geographical distributions (Figs. 2 and 4), the chloroplast haplotype groups of East Asian *Synstylae* species, particularly Sino-Japanese species (haplotype groups C–G), likely diversified following their migration and habitat fragmentation across the Sino-Japanese Floristic Subregion during the Pleistocene. Rejoining of the temperate forests of the Sino-Japanese Subregion during glacial periods of the Pleistocene may have allowed the migration and colonization of *Synstylae* populations with unique and rare chloroplast haplotypes, resulting in founder effects. Conversely, within fragmented and isolated temperate-forest populations divided by the East China Sea and Yellow Sea during interglacial periods, chloroplast haplotypes may have become fixed within specific haplotype groups via genetic drift (Harrison et al. 2001). Additionally, chloroplast DNA has a four-fold smaller effective population size than nuclear DNA due to its haploidy and uniparental inheritance, making it more susceptible to genetic drift (Blanchard and Lynch 2000; Palumbi et al. 2001). Compared to the diverse haplotypes of *R. multiflora* (haplotype groups C, E, and F) found in Sino-Japanese temperate forests, the unique haplotype of *R. transmorrisonensis* (group E) in Taiwan, which is possibly derived from *R. multiflora* through recent anagenesis (Jeon et al. 2025), exemplifies founder effects within the East Asian *Synstylae* (Figs. 3 and 4). Despite the distinct nuclear phylogenetic lineages of species found in the Korean-Manchurian region (*R. lucieae*, *R. maximowicziana*, and *R. multiflora*; Fig.S5; Jeon et al. 2025), the fixed presence of haplotype group E in this region supports the influence of genetic drift (Fig. 4). A prior population genetic study on another Sino-Japanese *Rosa* species, *Rosa rugosa* (sect. *Rosa*), also indicated possible founder effects and genetic drift in the Korean-Manchurian region (Xu et al. 2021). The non-significant Tajima’s *D* values across East Asian regions (− 2 ≤ *D* ≤ 2 or *p* > 0.01; see Table S7) corroborate the influence of genetic drift with neutral mutations rather than positive selection or selective sweep in each geographic region.

### Genetic introgression and chloroplast capture within the East Asian *Synstylae*

A prior phylogenetic analysis of East Asian *Synstylae* species using multiple nuclear orthologous markers delineated distinct species lineages and revealed their phylogenetic relationships independent of geographic distribution (Jeon et al. 2025). In contrast, the chloroplast haplotype phylogeny in the present study identified distinct haplotype groups that diverged along geographic gradients but did not resolve species-level relationships (Figs. 3 and 4; Fig S4). The substantial incongruence between the chloroplast and nuclear phylogenies, particularly among the Sino-Japanese *Synstylae* species, supports the hypothesis of chloroplast capture through genetic introgression, as suggested in previous studies (Fig. S5; Rieseberg and Soltis 1991; Wissemann and Ritz 2005; Zhu et al. 2015; Jeon and Kim 2019). Furthermore, multiple accessions of the same species from the same locality displayed conflicting haplotype groupings (e.g., haplotype groups C and E for Japanese *R. lucieae*, *R. multiflora*, and *R. onoei* var. *oligantha*; groups E and F for Chinese *R. multiflora*; and groups A and G for Japanese *R. sambucina*; see Fig. 4 and Fig. S5). Given contemporaneous divergence of chloroplast haplotypes and species lineages during the Pleistocene coupled with geographic intricacy of East Asia, an ancestral geographic isolation may have led the nuclear and chloroplast genomic differentiation of populations of East Asian *Synstylae* species. Following the probable gene flow after recurrent isolation and rejoining of these populations by climate oscillations, the nuclear DNAs and phenotypic characteristics of each East Asian *Synstylae* species lineage may have been recovered by backcrossing after genetic introgression, whereas the introgressed chloroplast haplotypes remain captured without recombination due to its uniparental inheritance.

The genetic drift of chloroplast haplotypes inferred within the Sino-Japanese *Synstylae* can result either from independent genetic drift within populations of each species or from shared genetic drift across sympatric or adjacent populations of multiple species over species boundaries. Although accessions of the same species from different localities were well clustered into monophyletic or paraphyletic groups in the nuclear phylogeny (Jeon et al. 2025), their chloroplast haplotypes were highly divergent in the chloroplast phylogeny (Fig. S5). Considering the divergence of robust species lineages within the Sino-Japanese *Synstylae*, independent genetic drift of different species populations without genetic introgression would have led the species-level divergence of each chloroplast haplotype after the divergence of haplotype groups. However, chloroplast haplotypes were shared by different species but not diverging into species lineages, particularly within haplotype groups C and E (Fig. 3; Fig. S4), supporting shared genetic drift over species boundaries within the Sino-Japanese *Synstylae*. Moreover, accessions of *R. maximowicziana*, one of the early diverging Sino-Japanese *Synstylae* species (estimated divergence time: 1.69 Mya; Jeon et al. 2025), particularly shared recently diverged haplotypes in group E (estimated crown age: 0.13 Mya; Fig. 2). This finding supports the probable genetic drift within *R. maximowicziana* after introgression of chloroplast haplotypes from recently diverged species lineages such as *R. multiflora* (estimated divergence time: 0.88 Mya; Jeon et al. 2025). Consequently, shared genetic drift of chloroplast haplotypes over species boundaries can provide strong evidence of prevalent genetic introgression within the Sino-Japanese *Synstylae* lineage (Figs. 3 and 4). Similar to the Sino-Japanese *Synstylae*, the interspecific chloroplast haplotype sharing along geographic patterns was investigated in several groups of closely related flowering plants in East Asia including the genera *Quercus* (Fagaceae; Li et al. 2022) and *Juglans* (Juglandaceae; Bai et al. 2010).

Chloroplast phylogenomic analysis has corroborated the putative hybrid origins of *R. lichiangensis* (Zhu and Gao 2015) and *R. pricei* (Jeon et al. 2025). Zhu and Gao (2015) proposed a hybrid origin for *R. lichiangensis* from maternal *R. soulieana* and paternal *R. multiflora* var. *cathayensis* based on morphological traits and genetic data from four chloroplast-intergenic regions and one nuclear region. Our comprehensive chloroplast genomic phylogeny aligns with this hypothesis, supporting the maternal contribution from *R. soulieana* to *R. lichiangensis*, as evidenced by their sister relationship within haplotype group A (Fig. 1). Jeon et al. (2025) proposed a hybrid origin of *R. pricei* with a uniparental contribution from *R. taiwanensis*, based on nuclear phylogenetic analyses. The close relationship between *R. pricei* and *R. taiwanensis* within chloroplast haplotype group D in our study suggests a probable maternal contribution from *R. taiwanensis* to the hybrid origin of *R. pricei* (Fig. 1; Fig. S4). Additionally, the incongruent phylogenetic positioning of these species between the uniparental chloroplast haplotype phylogeny and biparental nuclear ortholog phylogeny further supports the hybrid origin of *R. pricei*, with a potential paternal contribution from a distinct lineage outside *R. taiwanensis* (Fig. S5; Rieseberg and Soltis 1991; Jeon et al. 2025). These distinct hybrid origins underscore the significant role of genetic introgression within the East Asian *Synstylae* lineage, which fosters species diversification through the reticulation of species lineages.

## Conclusions

Chloroplast genomic phylogeny and haplotype analyses of recently diverged and closely related East Asian *Synstylae* species have shed light on the evolutionary history of chloroplast genome divergence along geographic gradients within the Eastern Asiatic Floristic Region. Founder effects, genetic drift, and introgression associated with migration and isolation may have influenced the divergence and diversification of chloroplast genomes within the East Asian *Synstylae* lineage, particularly in the Sino-Japanese Subregion. The incongruence between the chloroplast and nuclear phylogenies supported the prevalence of genetic introgression across the Sino-Japanese *Synstylae* lineage and postulated the existence of two putative hybrid species within this group. This study substantiates the important role of gene flow in plant diversity in East Asia and demonstrates the value of chloroplast genomes in uncovering evolutionary histories that may be obscured in the biparental nuclear genomes of closely related species. Furthermore, we highlight the significance of subtropical and temperate forests in the Sino-Japanese Floristic Subregion for maintaining plant genetic diversity, enhanced by geographical and climatic variability and the recurrent fragmentation and coalescence of populations (Harrison et al. 2001).

## Electronic supplementary material

Below is the link to the electronic supplementary material.

Supplementary Material 1

Supplementary Material 2

## Data Availability

The datasets generated and analyzed in this study are available in the NCBI GenBank, and their accession IDs are provided in the Supplementary Materials.
