# Supplementary material for: Chloroplast genome and haplotype relationships unravel the genetic introgression and complex evolutionary history of East Asian Rosa section Synstylae roses (Rosaceae)
Source: Bot Stud. 2025 Jun 13;66:17. doi: 10.1186/s40529-025-00466-y (PMC12165924; doi:10.1186/s40529-025-00466-y)
Supplement: Supplementary file 1 — Supplementary Material 1 [file 40529_2025_466_MOESM1_ESM.doc]

**Chloroplast genome and haplotype relationships unravel the genetic introgression and
complex evolutionary history of East Asian *Rosa* section *Synstylae* roses (Rosaceae)**

**SUPPLEMENTARY INFORMATION**

Supplementary data are available online and consist of the following:

**Fig. S1.** Map of Eastern Asiatic Floristic Region and its subregions in East Asia.

**Fig. S2.** Chloroplast genome map of the five East Asian *Rosa* sect. *Synstylae* species.

**Fig. S3.** Haplotype marker-candidate protein-coding genes and their number of parsimony-informative sites and nucleotide diversity in protein-coding sequences.

**Fig. S4.** Maximum-likelihood phylogenetic tree of chloroplast haplotypes of the East Asian *Synstylae* inferred from the four highly variable genic regions.

**Fig. S5.** Comparison of tree topologies of the chloroplast haplotype phylogeny and nuclear ortholog phylogeny.

**Table S1.** List of the East Asian *Rosa* sections *Synstylae* and *Chinenses* taxa analyzed in this study.

**Table S2.** Sample information of the East Asian *Rosa* sect. *Synstylae* accessions employed for the haplotype analyses in this study.

**Table S3.** Chloroplast genome accessions of *Rosa* sect. *Synstylae* species and their close relatives employed in this study.

**Table S4.** Statistics of five chloroplast genomes and their annotations of East Asian *Rosa* sect. *Synstylae* species assembled in this study.

**Table S5.** Marker information of the four highly variable chloroplast-genic regions for haplotype analyses in this study.

**Table S6.** Summary of the samples of the parameter values, effective sample sizes, and potential scale reduction factors in the Bayesian phylogenetic inference.

**Table S7.** Statistics of *Rosa* sect. *Synstylae* chloroplast haplotypes in East Asian regions.


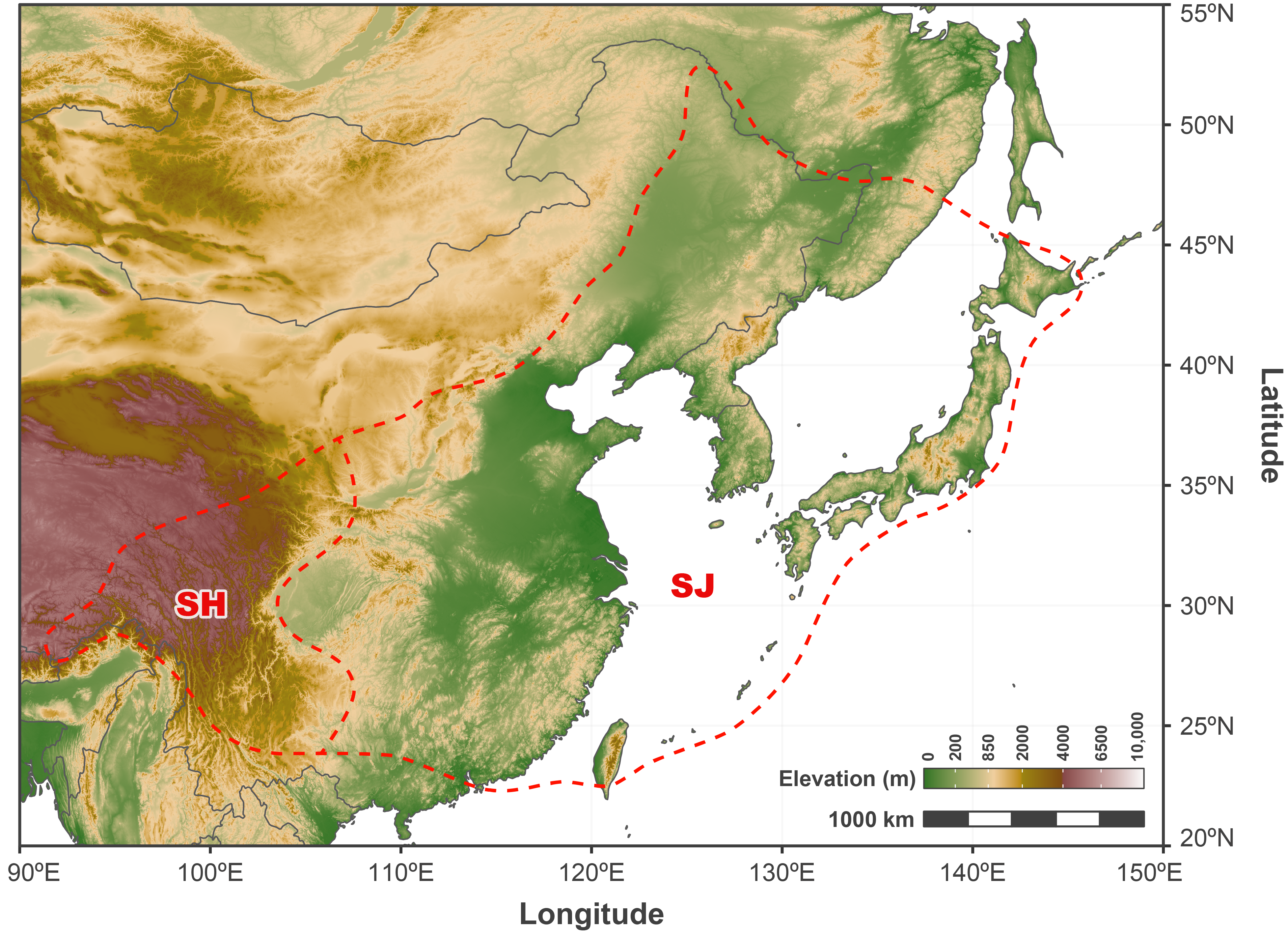


**Supplementary Fig. S1.** Map of the Eastern Asiatic Floristic Region and its subregions in East Asia, modified from Takhtajan (1978) and Qiu et al. (2011). SH stands for the Sino-Himalayan Subregion, and SJ stands for the Sino-Japanese Subregion.


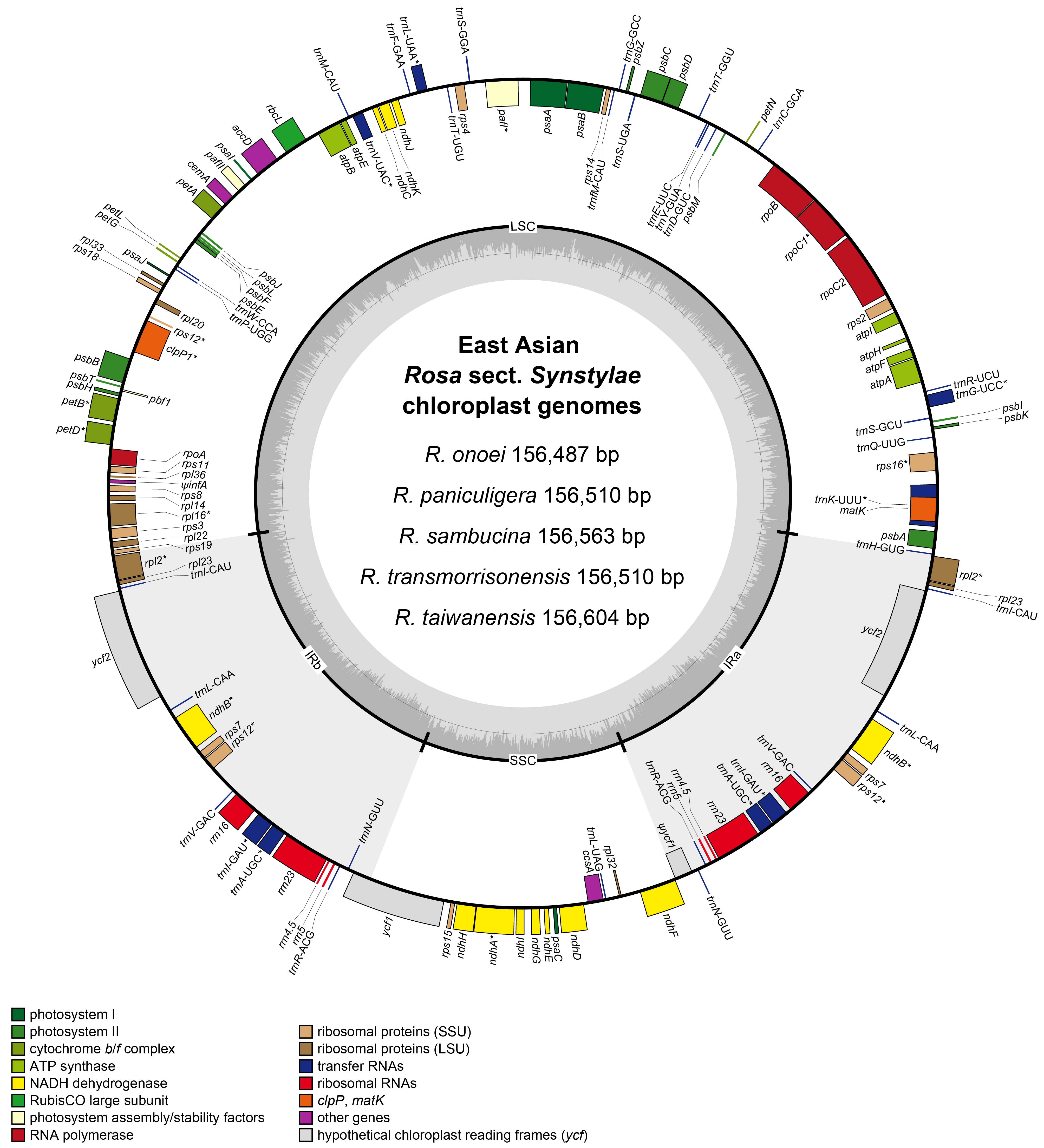


**Supplementary Fig. S2.** Chloroplast genome map of the five East Asian *Rosa* sect. *Synstylae* species. The gene regions were delineated on the outer circle, of which genes on the outside are transcribed in the counterclockwise direction and those on the inside are transcribed in the clockwise direction. The inner circle indicates the range of the large single-copy region (LSC), the small single-copy region (SSC), and two inverted repeats (IRs), and also shows a GC-content graph of the genome, where the dark-gray bars indicate GC content, while light-gray bars indicate AT content at each locus. The gray blocks between the outer and inner circles represent inverted repeat regions.


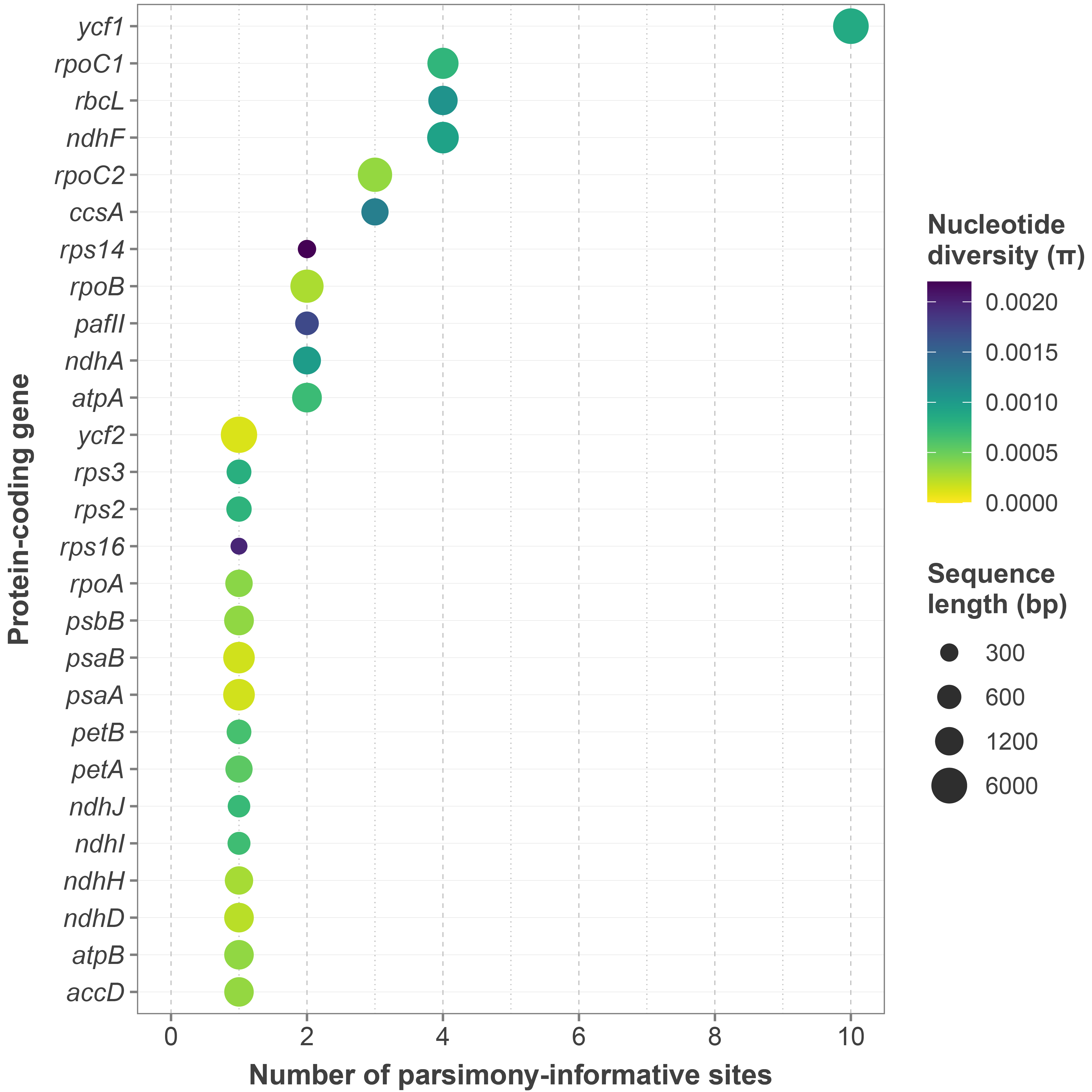


**Supplementary Fig. S3.** Haplotype marker-candidate protein-coding genes and their number of parsimony-informative sites and nucleotide diversity (π) in protein-coding sequences, within the sequence alignment of closely related Sino-Japanese series *Multiflorae* species.


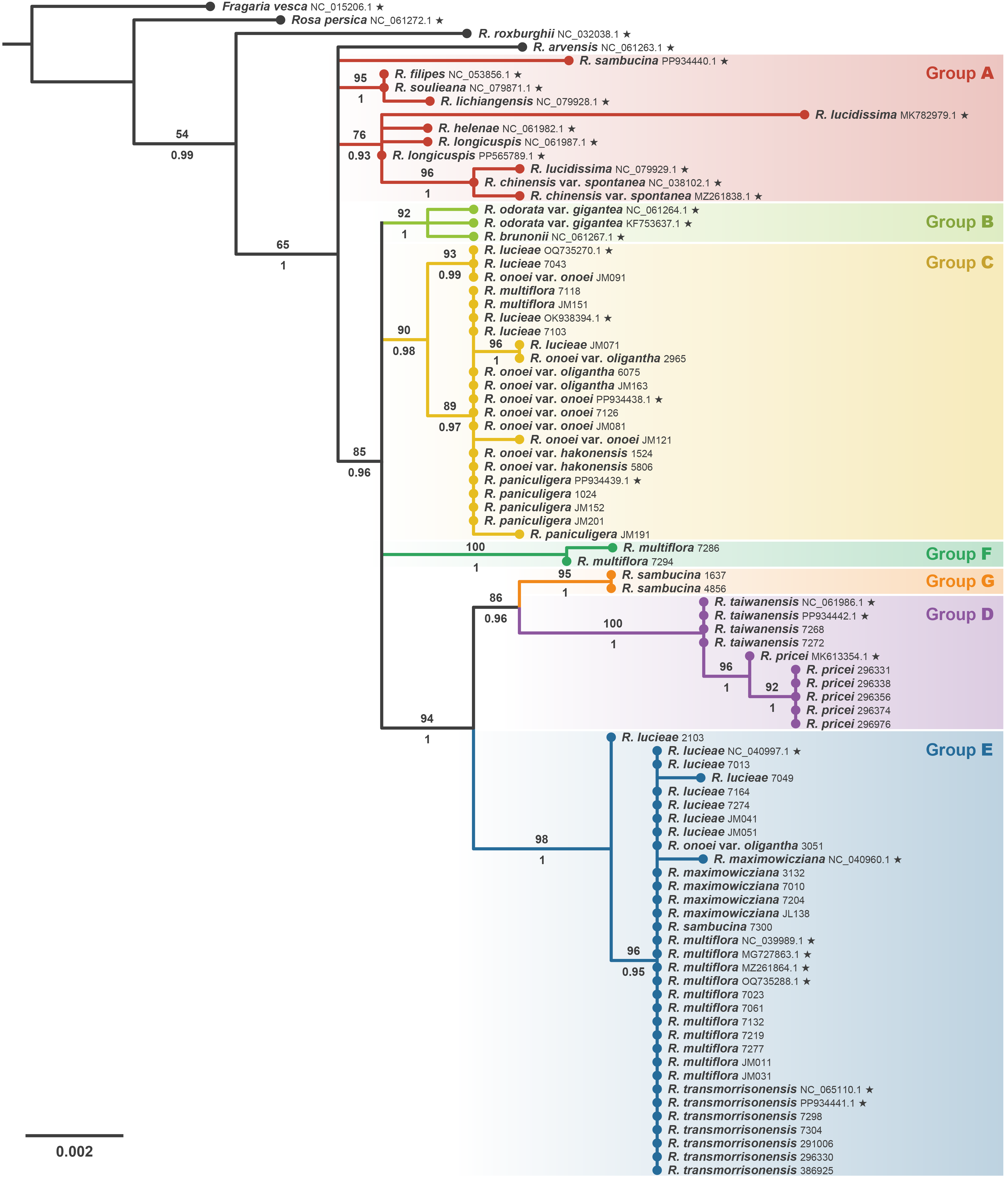


**Supplementary Fig. S4.** Maximum-likelihood phylogenetic tree of chloroplast haplotypes of the East Asian *Synstylae* inferred from the four highly variable genic regions. Numbers above branches indicate the bootstrap branch support, and numbers below branches indicate the posterior probability of Bayesian inference. Tip labels are species names of accessions, followed by accession IDs. Colors of branches, tips, and tip-label blocks represent the chloroplast haplotype groups. Accessions marked with stars indicate the chloroplast genomic accessions.


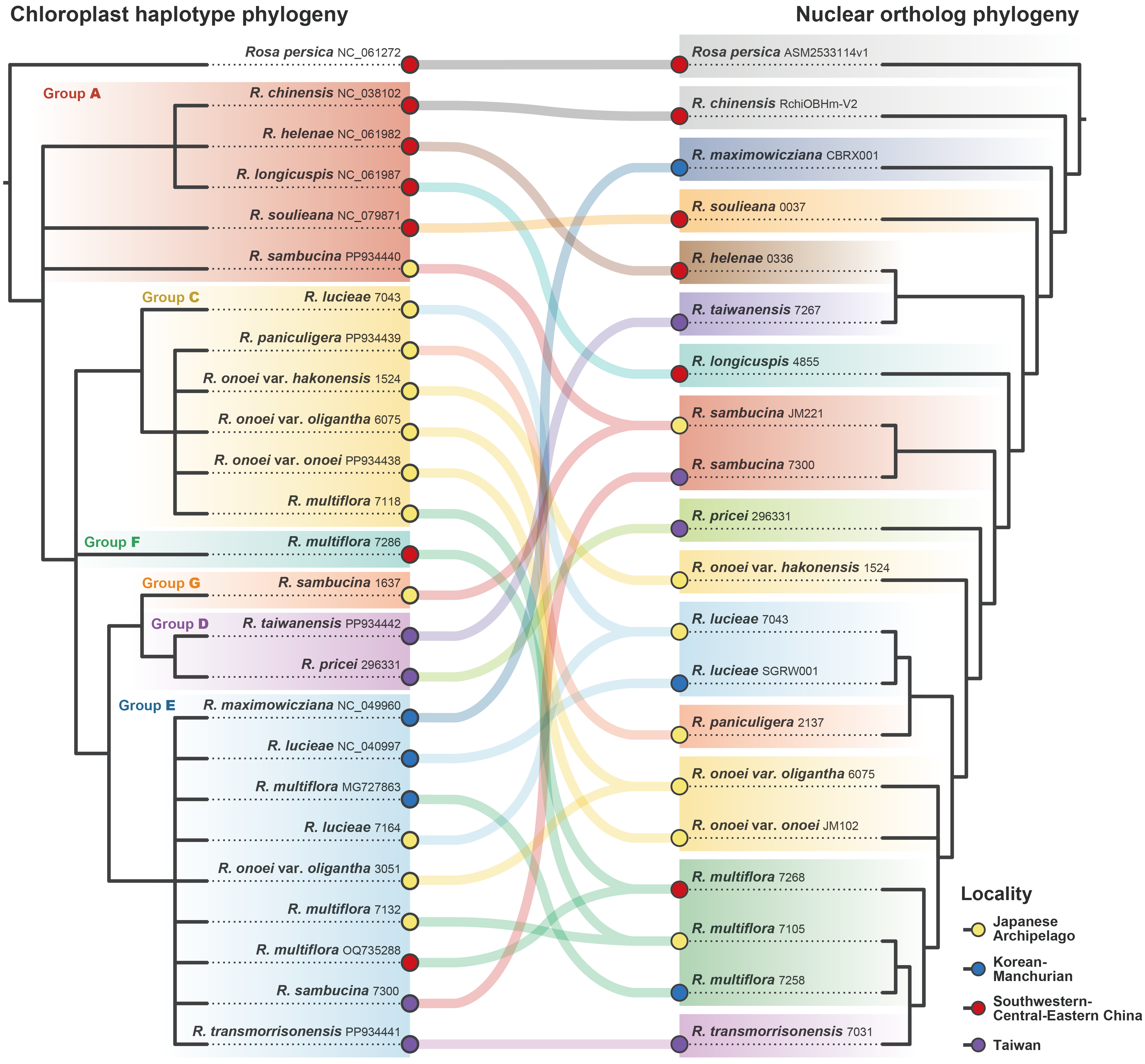


**Supplementary Fig. S5.** Comparison of tree topologies of the chloroplast haplotype phylogeny (left; the present study) and nuclear ortholog phylogeny (right; modified from Jeon et al., 2025). Tip labels are species names of accessions, followed by accession IDs. Tip colors represent the localities of accessions. Colors of tip-label blocks represent the chloroplast haplotype groups in the chloroplast haplotype phylogeny (left), and species in the nuclear ortholog phylogeny (right). Lines between two phylogenetic trees connect the accessions of same species and from same localities.
